# Two Different Maintenance Strategies in the Hospital Environment: Preventive Maintenance for Older Technology Devices and Predictive Maintenance for Newer High-Tech Devices

**DOI:** 10.1155/2016/7267983

**Published:** 2016-03-27

**Authors:** Mana Sezdi

**Affiliations:** Department of Biomedical Device Technology, Istanbul University, 34320 Istanbul, Turkey

## Abstract

A maintenance program generated through the consideration of characteristics and failures of medical equipment is an important component of technology management. However, older technology devices and newer high-tech devices cannot be efficiently managed using the same strategies because of their different characteristics. This study aimed to generate a maintenance program comprising two different strategies to increase the efficiency of device management: preventive maintenance for older technology devices and predictive maintenance for newer high-tech devices. For preventive maintenance development, 589 older technology devices were subjected to performance verification and safety testing (PVST). For predictive maintenance development, the manufacturers' recommendations were used for 134 high-tech devices. These strategies were evaluated in terms of device reliability. This study recommends the use of two different maintenance strategies for old and new devices at hospitals in developing countries. Thus, older technology devices that applied only corrective maintenance will be included in maintenance like high-tech devices.

## 1. Introduction

Medical technology includes all medical equipment used by health organizations for diagnosis, therapy, monitoring, rehabilitation, and care. Therefore, medical technology management plays a key role in health care. Effective medical device management is required to ensure high-quality patient care [1, 2]. Efficient and accurate equipment provides a high degree of patient safety. Accomplished medical device management will greatly assist in the reduction of adverse incidents and medical device-related accidents. For medical technology management, hospitals must have activities for maintaining, inspecting, and testing all medical equipment in the inventory. These activities must be performed within the scope of a program called “maintenance program.” The Medicine and Healthcare Products Regulatory Agency declares that maintenance activities and their intervals should be planned in accordance with the manufacturers' recommendations or strategies listed in an alternative equipment maintenance program [3]. These alternative program strategies must be based on valid standards of practice.

A maintenance program, generated by considering the characteristics and failures of medical equipment, is important with regard to usability and efficiency. However, it is inefficient to use the same strategies for the management of older technology devices and newer high-tech devices because of their different characteristics. The new high-tech devices functional control activities planned in accordance with the manufacturers' recommendations and daily programmed self-tests should be done. These devices are tested against their specifications presented by their manufacturers. According to the 2007/47/EC Directive, these tests must be planned by the manufacturers. The directive states that “The instructions for use must contain details of the nature and frequency of the maintenance and calibration needed to ensure that the devices operate properly and safely at all times” [4]. For this reason, daily checks, including visual controls and specific device tests, are described in the user guide and carried out by users.

Unlike new high-tech devices, the manufacturers' recommendations for older technology devices are not applicable because of the long usage time and device age. Generally, in developing countries, such as Turkey, older technology equipment mainly receives corrective maintenance. For example, a device is repaired when damaged or nondurable parts are replaced. In other words, maintenance is not specific to each device. Yearly maintenance contracts with manufacturers are only set for high-tech devices. This study investigated whether older technology devices could be included in maintenance strategies similar to those used for high-tech devices.

The quality of older technology medical devices can be ensured through periodical performance verification and safety testing (PVST) in accordance with international standards. PVST uses a standard measurement system with known accuracy to measure the accuracy of medical equipment [5–7]. PVST which includes qualitative and quantitative tests is performed by qualified biomedical personnel. During PVST, if a device is identified as not compatible with international standards, the hidden failures are determined and recorded by the biomedical staff. These failures are repaired by the hospital's biomedical staff or service technicians employed by manufacturers. This process discloses the possible failures of medical equipment.

All test results indicate causation, a tremendously important factor in the prevention of adverse incidents and generation of an effective maintenance program. Valuable lessons can be learned from an analysis of failures and these can be applied to maintenance programs [6]. Therefore, failure analysis is the main activity of a maintenance program.

Recently, the demand for medical device management is increasing as the number of medical devices increases. Therefore, the development of more effective maintenance programs has achieved prominence.

The initial purpose of this study was to generate a maintenance program comprising two different maintenance strategies, one each for older technology devices and newer high-tech devices, utilizing the manufacturers' recommendations and PVST results, respectively, and to determine the success rate of this program using the indicators. Accordingly, old technology devices will be included in the maintenance systems through separate testing.

The Food and Drug Administration maintains a database of medical equipment failures and has conducted some preventive studies for all medical sectors [8]. Several reports have analyzed medical failures and preventive maintenance [9–20]. However, very little information about hidden failures was available in the literature when the collection of hidden failure data was initiated for this study. Wallace and Kuhn presented an analysis of software-related medical device failures that led to manufacturer recalls, but they caused no deaths or injuries [9]. In addition, Bliznakov et al. reported medical device recalls due to software failures. The authors collected data related to software failures and performed an analysis via failure classification [10]. Many other studies have presented alternative maintenance strategies for each piece of equipment. Ridgway et al. classified failures in an attempt to reduce equipment downtime [11]. Santos and Almeida prepared maintenance schedules using mean intervals between failures [12]. Taghipour et al. studied a multicriteria decision-making model to prioritize medical devices according to criticality [13]. Taghipour et al. also described a periodic inspection optimization model for complex repairable systems [14, 15]. Hamdi et al. presented a new approach to work-order prioritization for medical equipment maintenance requests [16]. Taghipour and Banjevic modeled an optimal periodic inspection interval in a preventive maintenance [17]. Khalaf et al. presented evidence-based maintenance using a mixed integer model [18]. Miniati et al. analyzed the technical data from medical devices with support from technology managers [19]. Lastly, Saleh et al. used quality function deployment to solve problems related to preventive maintenance prioritization [20]. The present study differs from previous studies because it presents a maintenance program created using two different strategies. The first strategy incorporates daily checks for new high-tech devices, whereas the second implements PVST as the sole performance measurement for older technology devices. To the best of our knowledge, no previous study has focused on hidden medical device failures determined during the PVST of medical devices.

## 2. Methods

**Figure 1:**
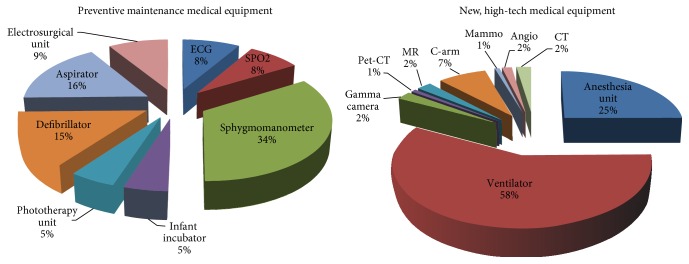
Medical devices in the old technology and new high-tech device groups.

This study included a total of 723 high-risk medical devices maintained by the Medical Faculty of Istanbul University [21]. Low-risk devices were excluded from the study. The high-risk devices were classified as older technology devices and newer high-tech devices. This classification was performed because of the lack of service or user manuals, and corresponding lack of manufacturer recommendations for many older technology devices. This lack makes it impossible to apply the same procedures to old technology and new high-tech devices. Thus, different procedures were applied to old technology and new high-tech devices in order to develop maintenance. As seen in Figure 1, old technology devices (589 devices) comprised of electrocardiography (ECG) devices, pulse oximetry devices, sphygmomanometers, infant incubators, phototherapy units, defibrillators, surgical aspirators, and electrosurgical units. These devices were tested by applying PVST. The second group (134 devices), which mostly contained imaging devices, comprised computerized tomography (CT), angiography, mammography, C-arm radiography, magnetic resonance (MR), and positron emission tomography and gamma cameras. The second group also included ventilators and anesthesia devices used in intensive care departments. Accordingly, these devices had a 24-hour workload. The devices in the first group, excluding those used in intensive care and emergency departments, had 8-hour workloads. The groups were investigated separately and two different maintenance strategies were developed: predictive maintenance for newer high-tech devices and preventive maintenance for older technology devices.

The development of preventive maintenance for older technology devices required a long procedural duration, whereas predictive maintenance for newer high-tech devices was developed in accordance with the manufacturers' recommendations.

### 2.1. Predictive Maintenance for Newer High-Tech Devices

A predictive maintenance program for newer high-tech devices was developed by applying maintenance time schedules created according to the manufacturers' recommendations. Under this program, predictive maintenance was conducted for each device through a contract with the manufacturer's technical service and cooperation of the biomedical department of the hospital. The hospital's biomedical personnel also attended the maintenance activities. After each service session, maintenance reports were delivered to the biomedical staff by the manufacturer's technical service department.

Users performed daily checks of the devices. They were trained in the performance of daily checks through user training provided by the manufacturer. Users reported failures identified during daily checks. The most important point in this section was the collection of regular feedback from all device users. Training aimed to ensure that the smallest failure would be reported. Although the exact training success and feedback rates were not determined, an increase in feedback was observed. In addition, failures occurring during work hours were reported to the manufacturer's technical service by the hospital's biomedical personnel. Staffs also attended to failure detection and provided failure reports. As a result, data were obtained and used to evaluate predictive maintenance.

### 2.2. Preventive Maintenance for Older Technology Devices

A preventive maintenance program for older technology devices was developed via analysis of the PVST results of the equipment. The following PVST steps were performed in sequence by the hospital's biomedical personnel [5]:

- determination of the PVST intervals,
- application of the PVST,
- interpretation of the PVST results according to the acceptance criteria stated in international standards.

#### 2.2.1. PVST Intervals

PVST intervals were determined by calculating the Equipment Management Number (EMN), which is described in the Clinical Equipment Management standards of the Technology and Safety Management series developed by the Joint Commission. Given the lack of the manufacturers' recommendation for the old technology devices investigated in this study, the EMN was used with a general approach to determine the initial PVST interval. PVST intervals accepted by industry could have been used if the manufacturers' recommendations were present [22]. The EMN technique, introduced by Fennigkoh and Smith, classifies equipment using three parameters: function, risk, and maintenance requirements [23]. A numerical value is assigned to each device type by classifying the above-mentioned parameters. The scores used to calculate EMN can be seen in Table 1. Specifically, the EMN is the sum of the Equipment Function Score, Equipment Risk Score, and Maintenance Requirement Score. PVST intervals range from 6 to 12 months, depending on the EMN. According to the standards, EMN can have a maximum value of 20. If the calculated number is 12 or higher, the equipment is incorporated into the annual PVST plan. In addition, if the EMN is greater than 17, the device must be controlled every 6 months.

The calculated EMN and PVST intervals determined for the medical equipment investigated in this study are shown in Table 2.

#### 2.2.2. PVST Procedures

PVST was performed according to the procedures of Inspection and Preventive Maintenance (IPM), prepared by the Emergency Care Research Institute [24]. In this study, testing parameters for medical devices measured during PVST were determined from IPM procedures. The procedures comprised both quantitative and qualitative tests. The qualitative test evaluated the device's physical parameters (e.g., connectors, battery, and electrodes). The quantitative test includes functional controls. The main principle was the evaluation of all functional parameters of the medical device. Although the qualitative test was general, the quantitative test was specific for each device. The quantitative test parameters measured for each device in this study are listed in Table 2.

Tests were performed with a low level of uncertainty, which was calculated using the procedures declared in the Guide to the Expression of Uncertainty in Measurement (GUM).

#### 2.2.3. Interpretation of the PVST Results

To interpret the PVST results, the acceptance criteria in the IPM procedures were considered. Medical devices for which measurement results fell within the acceptable range were considered appropriate with respect to international standards and were labeled with green stickers. This designation indicated that the medical equipment passed the inspection and could be used. Test results were accordingly recorded as “Passed” (P) in documentation and the database. Medical devices for which measurement results fell outside of the acceptable range were considered inappropriate with respect to international standards and were labeled with red stickers. The red sticker indicated the presence of failures and stated that the device should not be used. The corresponding PVST result was recorded as “Failed” (F) in documentation and the database.

Clinical staffs were trained with regard to their responses to each label color. According to the procedure applied for red label devices, staffs were prohibited from using the device, which was sent to the clinical engineering department to identify and remove any hidden failures that did not completely disable the primary device functions. After correction and a second PVST, the device could be returned for use in the department.

All data regarding information about the equipment, such as the equipment name, location, serial number, interpretation results (Passed or Failed), and failure definition, were entered in the operation page. A sample page is shown in Table 3.

PVST, when performed at a determined interval, provides a large statistical failure dataset that could be used to establish a maintenance interval. The preventive maintenance time schedule was planned using data obtained from PVST results. Hidden failures detected during PVST that could affect device performance were considered during preventive maintenance planning. For example, a 3-month interval was planned for maintenance of the most common hidden failures detected in incubators during PVST. A 6-month interval was planned for maintenance of less frequently encountered failures. Maintenance checklists were prepared and required nondurable parts for continuous medical equipment service were determined. The maintenance process with regard to the qualitative and quantitative device parameters was defined using maintenance checklists. The defined processes included control, cleaning, calibration, replacement, and measurement. The checklist stated which part was to be subjected to which process. Notably, the same part may include more than one process. For example, batteries are initially checked and subsequently changed. Similarly, pedals are controlled, cleaned, and changed if necessary.

### 2.3. Evaluation of the Maintenance Program

The performance of maintenance strategies for older technology devices and newer high-tech devices was assessed in terms of progress in achieving the expectation defined by the program. Maintenance activities were evaluated using a failure rate indicator. A 6-month validation phase was planned to monitor whether the failure rates of old technology devices and new high-tech devices would decrease with the application of the maintenance plan. This phase was selected because defibrillators and electrosurgical units selected pilot devices for the evaluation of the preventive maintenance and have a 6-month PVST interval.

No preventive maintenance was conducted in the hospital before this study. However, medical devices were subjected to PVST before the study. The hidden failures of all medical devices were recorded. To evaluate preventive maintenance, the results of PVST during preventive maintenance were compared with the results of PVST before maintenance. In addition, the predictive maintenance results were evaluated by comparing the failures that occurred within 6 months of prepredictive maintenance and those that occurred during predictive maintenance. Data of failures that occurred before predictive maintenance were extracted from each device's failure history which was available in the hospital documentation.

A reporting system was planned in which an archive of all devices' failure histories would be created. This reporting system enabled the monitoring of all medical device information. To this end, biomedical personnel collected user checklists and technical service forms from the manufacturers' technical services. Data in these documents were entered on device information cards to generate a failure history for each device. Hence, this method provided a reporting system comprising the collection of data from checklists. Parameters related to failures, such as the failure definition, repair time, and replaced parts, were followed easily. In particular, unwanted data, such as the maximum repair time and more frequent failure rate, were identified. These data were reported to the decision-maker to explain the overall situation. For this, a one-page report was designed to supporting decision-makers in the allocation of an increased budget for technology procurement, new maintenance contracts, or more biomedical personnel. A sample report form for decision-makers is shown as follows: 

*Executive Summary*

*Medical Devices Predictive and Preventative Maintenance Report*

 Number of Total Medical Device:
 Number of Total Medical Device subject to Preventative Maintenance:
 Number of Total Medical Device subject to Predictive Maintenance:

(1) Failed devices often

 Location
   —
   —
 Device
   —
   —
 Manufacturer
   —
   —
 Model
   —
   —
 Serial Number
   —
   —

(2) The failed devices in the warranty period

 Location
   —
   —
 Device
   —
   —
 Manufacturer
   —
   —
 Model
   —
   —
 Serial Number
   —
   —

(3) Failed end-of-support devices

 Location
   —
   —
 Device
   —
   —
 Manufacturer
   —
   —
 Model
   —
   —
 Serial Number
   —
   —

(4) Parts which were need to be replaced but not included in the annual maintenance contract

 Location
   —
   —
 Device
   —
   —
 Manufacturer
   —
   —
 Model
   —
   —
 Serial Number
   —
   —

(5) Devices which could not be found their non-durable parts and could not be repaired

 Location
   —
   —
 Device
   —
   —
 Manufacturer
   —
   —
 Model
   —
   —
 Serial Number
   —
   —

(6) Non-durable parts which have been changed again in the warranty period although they had been changed before

 Location
   —
   —
 Device
   —
   —
 Manufacturer
   —
   —
 Model
   —
   —
 Serial Number
   —
   —

(7) The devices which can not get technical service from their contracted firm

 Location
   —
   —
 Device
   —
   —
 Manufacturer
   —
   —
 Model
   —
   —
 Serial Number
   —
   —

This report will help decision-makers to define short- and long-term priority plans for technology investments based on safety aspects.

## 3. Results

This study planned predictive maintenance for 134 newer high-tech devices and preventive maintenance for 589 older technology devices.

### 3.1. Predictive Maintenance

Planned predictive maintenance involved the usage of daily checklists created by the manufacturers. These daily checklists which included simple, mandatory pre- and post-use tasks, were presented to the device users. Maintenance time schedules were planned according to the manufacturers' recommendations provided in the user guides.  Table 4 shows a sample maintenance time schedule, including each device brand and model. The devices were also subjected to monthly maintenance, in addition to the recommended maintenance period. Since annual maintenance fee payments are divided into 12 months in accordance with the Turkish currency system, the contracted company must visually maintain the device every month. This visual maintenance comprises short-term maintenance, especially device cleaning.

### 3.2. Preventive Maintenance

**Figure 2:**
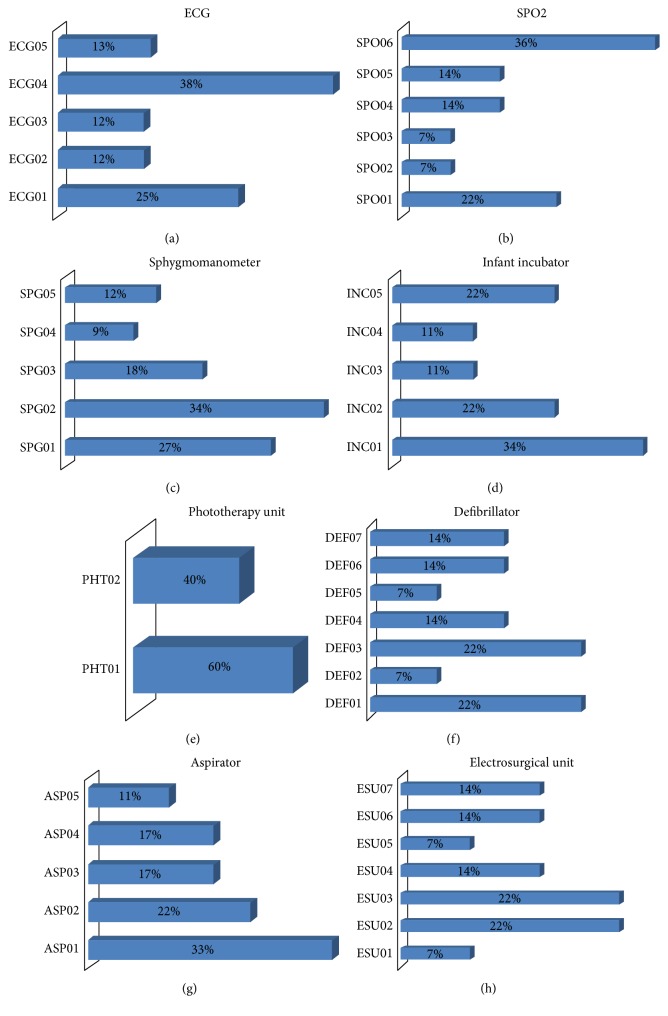
Failures specific for each type of older technology medical device.

For older technology devices, preventive maintenance was planned by analyzing PVST results. Accordingly, failures were detected in 126 (22%) of the 589 medical devices from different departments in the Medical Faculty at Istanbul University; they were marked as “Failed,” and the remaining 463 were marked as “Passed.” When the “Failed” devices were analyzed according to their errors, several technical hidden failures were observed. The failures are summarized in Table 5 according to error code. In addition, Figure 2 presents the distribution of hidden failures.

PVST results were used to plan a preventive maintenance time schedule for old technology devices. This preventive maintenance time schedule indicates the maintenance interval for the device. The adequate interval for effective maintenance was determined for each device and nondurable part. Equipment with recorded failures was assigned a more frequent maintenance schedule. The time schedules for old technology devices are shown in Table 6.

Preventive maintenance checklists were prepared for the devices. These checklists defined the maintenance process of the qualitative and quantitative device parameters. The checklist also stated which part was to be subjected to which process. The required visual, functional, and electrical controls were explained in the checklist. In addition, nondurable parts requiring replacement were identified in the checklist.

The PVST analysis revealed that nondurable parts (e.g., ECG patient electrodes, oximeter probes, cuffs, defibrillator batteries, and ultraviolet lamps) must be stocked for each piece of medical equipment. Both the number and features of the nondurable parts required for each type of medical device were determined. However, spare parts were not stocked for devices maintained by manufacturers' technical services.

### 3.3. Performance of the Maintenance Program

The primary focus of preventive and predictive maintenance is the reliability of medical devices [25]. Therefore, reliability was analyzed to evaluate the performance of preventive and predictive maintenance. Indicators such as failure rates per old technology devices and per new technology devices were determined to evaluate the equipment reliability.

**Figure 3:**
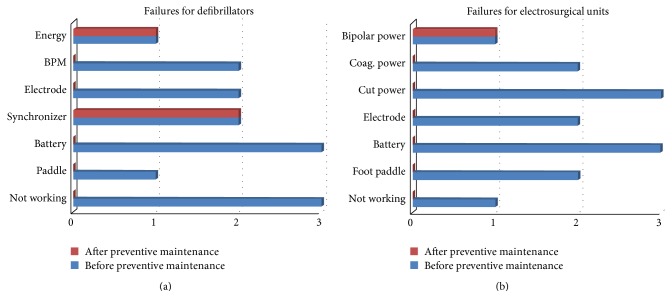
(a) Failures of the defibrillators and (b) of the electrosurgical units before and after preventive maintenance.

The success of preventive maintenance was evaluated by analyzing the results of PVST performed after preventive maintenance. The results of PVST performed before and after preventive maintenance were then compared. Since preventive maintenance includes old technology devices, the failure rate of sample devices provides information about the failure rate of old technology devices. The defibrillator and electrosurgical units were selected as pilot medical devices. Before preventive maintenance, 86 defibrillators and 52 electrosurgical units were inspected, and their results were analyzed to develop preventive maintenance. After maintenance, the devices were inspected again after a period of 6 months. Figures 3(a) and 3(b) show the results of the qualitative and quantitative tests performed before and after preventive maintenance, respectively. In addition, PVST results obtained after preventive maintenance indicated that the minor and major defects detected during PVST were largely rectified. Traditional preventive maintenance (TPM) of some components was also performed. For example, batteries were replaced before complete depletion, and paddles were lubricated to increase conductivity. Therefore, in contrast to the results of PVST performed before preventive maintenance, fewer failed components were identified after preventive maintenance.

The defibrillator is a high-risk device with regard to patient safety. The patient's life is at risk if the device fails completely or does not provide sufficient energy to the patient when in use. The parameters measured during PVST reflect the risks of the defibrillator. In particular, the quantitative parameters such as “output energy,” “charge time,” and “energy after 60 sec” are important parameters that may pose a serious threat to the patient. During the evaluation of preventive maintenance, the incidence of these failures was found to have decreased. The qualitative parameters of defibrillators are related to physical specifications. Although they are considered to have a lesser impact on patient safety, quantitative parameters are also important because they are directly related to defibrillator function. This is one reason why the defibrillator was used as a pilot device during preventive maintenance evaluation. All parameters affect defibrillator operation directly and patient safety indirectly. The above-described situation is also valid for the second pilot device, the electrosurgical unit. The other devices investigated in this study, such as ECG, pulse oximeter, and aspirator, might cause some inconvenience to the patient, but they do not pose a serious risk. In these devices, the determined failures were hidden and indicated deviations from the devices' functional performance specifications. Hidden failure repair is required to prevent serious failures and to ensure standard service from the device but is not essential for the patient safety.

For both pilot devices, problems related to quantitative parameters that were determined by PVST before preventive maintenance were resolved during preventive maintenance (Figure 3). The three nonworking defibrillators were restored to a working condition. Batteries, pedals, and electrodes with issues were changed. Only problems related to the synchronizers of two defibrillators could not be resolved by the hospital's biomedical staff and required manufacturer's technical service. In addition, an issue with the output energy of one defibrillator was also not resolved during preventive maintenance (Figure 3(a)) and the device was sent to the manufacturer's technical service. Similar to the defibrillators, all hidden failures in the electrosurgical units were resolved during preventive maintenance, except for a bipolar power-related problem (Figure 3(b)).

**Figure 4:**
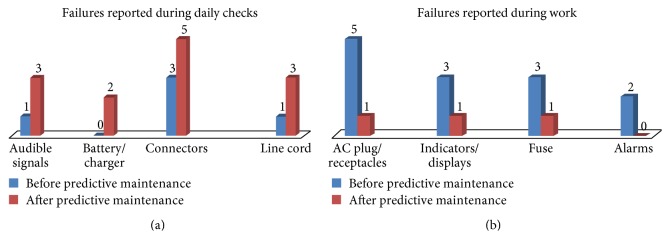
Failures of the gamma camera before and after predictive maintenance. (a) Failures reported during daily checks. (b) Failures reported during work.

**Figure 5:**
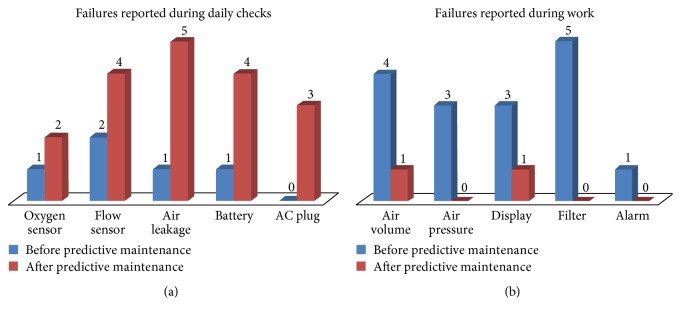
Failures of the ventilator before and after predictive maintenance. (a) Failures reported during daily checks. (b) Failures reported during work.

The success of predictive maintenance was evaluated by analyzing medical equipment failure reports after predictive maintenance. The gamma camera and ventilator were selected as pilot medical devices. Their failure rates could be assumed to represent the failure rate of new high-tech devices. Failures occurring within 6 months of pre- and postpredictive maintenance were extracted from the devices' failure histories, which were available in the hospital documentation. Figures 4 and 5 present the failures of gamma cameras and ventilators occurring pre- and postpredictive maintenance, respectively. The failures were classified into two categories: those reported during daily checks and those occurring during work. The latter group caused the devices to stop working. In contrast, the former group was generally noticed during daily checks and generally related to the device's physical condition or software. Accordingly, such failures had no or little influence on the device's functioning. As shown in Figures 4 and 5, a greater number of failures were reported during daily checks after predictive maintenance. This might be attributed to failures being ignored by users during daily checks prior to predictive maintenance. The increased reporting of failures during daily checks indicates the user support of predictive maintenance. In contrast, a greater number of failures occurring during work were reported before predictive maintenance. This indicates that some failures during work were prevented by applying predictive maintenance.

## 4. Discussion

This report describes the formation of a maintenance program using the PVST results for older technology medical devices and the manufacturers' recommendations for newer high-tech devices. The resulting maintenance program forms a basis for quality assurance practices.

This study differs from other studies reported in the literature on two points. The first point is that older technology devices and newer high-tech devices were investigated separately. This led to the use of two different methodologies: PVST for older technology devices and the manufacturers' recommendations for newer high-tech devices. It is important to overcome such failures before they occur and thus avoid harming the patient. Unresolved failures may lead to several types of important medical equipment accidents. Accordingly, use of the PVST results was preferred for the development of a maintenance program for older technology devices.

The second point is that this study examined hidden medical equipment failures arising from noncompliance with international standards. Although other studies investigated hardware or software failures of medical devices, the present study addressed hidden failures that affect the quality of the medical device and patient safety. Wallace and Kuhn [9] and Bliznakov et al. [10] also presented an analysis of failures. But, these failures were related to software and resulted in device recalls by the manufacturers. In the present study, hidden failures were analyzed to develop a preventive maintenance protocol, and failures detected during daily checks and usages were analyzed to develop a predictive maintenance protocol.

Ridgway et al. classified failures in terms of repair calls. According to the authors, calls related to failures were classified as follows [11]:

- user-related calls,
- accessory- or connectivity-related calls,
- physical-stress-related calls,
- environmental-stress-related calls,
- human-interference-related calls.

After classification, the authors recommended user training, a well-managed battery-care program, availability of the proper accessories, and maintenance of the environmental conditions specified by the equipment manufacturer. In this study, failures were classified and analyzed in terms of technology levels (older technology devices and newer high-tech devices). Since failures were detected through daily checks, the failures used to develop predictive maintenance were related to user competence, accessories, connectivity, or environmental stress. In addition, since hidden failures were detected during PVST, failures used to develop preventive maintenance were related to accessories, connectivity, and environmental stress. Therefore, it can be stated that both preventive and predictive maintenance require a plan for maintaining the availability of proper accessories, as recommended by Ridgway et al. However, in the present study, the battery-care program was included in accessories planning rather than as a separate plan. In addition, biomedical staff competence with regard to PVST is more important than user competence in our model.

Taghipour and Banjevic used the mean time between failures to prepare maintenance schedules. The authors determined the maintenance activity intervals of devices in terms of intensity of use. They reported that devices with a high intensity of use require maintenance more frequently than devices with a low intensity of use [17]. In the present study, the maintenance schedule was prepared in accordance to the rate of device failure. Devices with high failure rates were scheduled to receive more frequent maintenance.

The present study and a small component of a study conducted by Taghipour [25] are only similar in terms of the PVST results analysis. Taghipour analyzed the PVST results of infusion pumps before and after preventive maintenance and noted that medical devices included in a maintenance program have smaller errors than other devices. Similarly, as shown in Figures 3(a) and 3(b) in the present study, fewer hidden failures were identified after preventive maintenance. This was in contrast to the results of PVST performed before preventive maintenance.

As mentioned above, the present study included two different maintenance strategies: predictive and preventive maintenance. Programmed maintenance based on the manufacturers' recommendations for new high-tech medical devices represented the predictive maintenance strategy. Predictive maintenance is known as time-based maintenance and is defined as a maintenance strategy wherein maintenance activities are performed at scheduled time intervals recommended by manufacturers [25]. In contrast, programmed maintenance based on an analysis of the performance inspection results of old technology medical devices represented the preventive maintenance strategy. Preventive maintenance is also known as condition-based maintenance and is defined as a maintenance strategy that involves periodic and continuous equipment condition monitoring to detect equipment degradation [25]. The information obtained from PVST results was used to determine the maintenance requirements and maintenance time schedule. For example, decision regarding filter replacement before the manufacturer's recommended replacement interval was based on equipment PVST results.

Whereas the predictive maintenance strategy was applied to individual components of new high-tech devices in consideration of the equipment brand and model, the preventive maintenance strategy was applied to groups of equipment such as defibrillators and oximeters.

Maintenance programs require resources such as budgets and test equipment. Predictive maintenance requires a budget to facilitate contracts with the manufacturer technical services, whereas preventive maintenance requires sensors and special equipment to conduct PVST.

In the predictive maintenance program, the user is responsible for reporting problems. If the user reports a problem, it will be added to the database. However, biomedical staffs conduct the PVST of equipment included in a preventive maintenance program. The analyzed failures are directly related to the device. This does not cover faults caused by the users.

Predictive maintenance may not always be optimal. Since a time-based schedule is implemented a device may receive more or less maintenance than required. However, using the preventive maintenance program, it is possible to track medical devices' hidden failures and to determine the most appropriate maintenance in terms of required nondurable parts and elapsed time. The replacement of components with failures was included in this model, as devices with nondurable parts fail if those parts are not replaced or restored. Preventive maintenance activities were performed by the hospital's biomedical personnel. These personnel also replaced nondurable parts. Nondurable parts requiring replacement at regular intervals should be stocked to ensure uninterrupted maintenance. These parts are supplied with a storage period of 1 year. For nondurable parts that are replaced twice yearly, a stock of two should be kept and a stock of three should be kept for parts replaced thrice yearly. Otherwise, maintaining a supply of expired nondurable parts extends the maintenance process and disrupts preventive maintenance.

As mentioned above, the current program helps to prevent problems prior to medical equipment failure and to maintain a stock of required nondurable parts. This feature increases the performance and efficiency of biomedical staffs. Medical equipment can be better tracked by repeating PVST throughout the year, as the database of equipment failures will contain PVST histories of older technology devices.

Preventive maintenance involves relatively old technology. Since the number of older technology devices in a hospital is greater than the number of new high-tech devices, the scope of preventive maintenance is more extensive than that of predictive maintenance. The distribution of failures might change because some old devices will be removed from use. By adding new devices to the inventory, the scope of preventive maintenance will become narrower, and the scope of predictive maintenance will become broader. However, new high-tech devices are also more expensive than older technology devices. Accordingly, predictive maintenance will be more costly than preventive maintenance.

This study has some limitations, because it was limited to high-risk devices in terms of patient safety and cost. Devices that pose risks to patients and users, old devices, and complex devices such as radiology devices are frequently considered for maintenance. Accordingly, the study did not include low-risk devices such as nebulizers and flow meters. The scope of the study could be extended to include other high-risk devices. For example, anesthesia units and vaporizers might be included in the preventive maintenance category. Although all endoscopy systems, including colonoscopy, gastroscopy, bronchoscopy, and laryngoscopy devices, could be incorporated into a preventive maintenance strategy, they do not undergo PVST. Rather, these devices are controlled via fluid leakage tests after each use. If there is any leakage, corrective maintenance is implemented. Operating tables and electrical patient beds requiring only electrical safety measurements may be incorporated into preventive maintenance.

In addition, all newly acquired equipment will be included in a maintenance program after considering its technology level.

The other limitation of the proposed model is that the criteria suggested by Fennigkoh and Smith [23] were used to determine the PVST interval. The EMN was used because this parameter has been accepted as a supervision criterion by the Ministry of Health in Turkey.

This study predicted that the reliability and failure patterns of a device would be affected by external factors such as the expertise level of users and biomedical staffs. Accordingly, the users were trained in the performance of daily checks through user training provided by the manufacturer. In addition, the clinical staffs were also trained about their responses to the different colored label on the devices after PVST. Both types of training were important for successful maintenance.

## 5. Conclusion

This paper proposes two different maintenance strategies: preventive maintenance for old technology devices and predictive maintenance for new high-tech devices. The first strategy takes into account the results of performance verification and safety testing. The second strategy considers the manufacturer recommendations.

Although preventive and predictive maintenance strategies differ in many ways, a maintenance program comprising both strategies yielded positive results. The maintenance strategy evaluation demonstrated that strategies based on PVST results and the manufacturers' recommendations led to a significant reduction in equipment failures and a significant increase in corrective maintenance.

The usage of different maintenance strategies for older devices and newer high-tech technology devices to develop maintenance strategies is important in terms of its consequences.

Firstly, the older technology devices that applied only corrective maintenance in developing countries will be included in the maintenance strategies like newer high-tech devices.

Secondly, the inclusion of both old and new technology devices to the maintenance system provides a wider range of maintenance that covers all medical devices in hospitals with many old technology devices.

Thirdly, the performance verification and safety testing earn importance to develop maintenance strategies for devices without manufacturer recommendations.

Lastly, considering carefully all outcomes of the medical equipment failures and existence of a detailed history for every device help decision-makers to manage medical equipment.

The next plan is to continue the study of failures of other medical devices excluded from this initial study.

## Figures and Tables

**Table 1:** Scores used to calculate the Equipment Management Number.

Score	Function	Risk	Maintenance requirement
10	Life recovery	—	—
9	Surgical and intensive care	—	—
8	Physical therapy	—	—
7	Surgical and intensive care	—	—
6	Other physiological monitors	—	—
5	Analytical laboratory	Patient death	Very important
4	Laboratory equipment	Patient-staff injury	Moderately important
3	Computers	Wrong diagnosis	Less important
2	Belong to the patients	Treatment delays	The least important
1	Other equipment pieces	Risk not important	Minimally important

**Table 2:** PVST parameters and PVST intervals of medical devices (FP: function point, RP: risk point, MR: maintenance requirement, and EMN: equipment management number).

Medical device	PVST parameters	Simulator analyzer measurement device	FP	RP	MR	EMN	Test interval
Electrocardiography	Linearity sensitivity 1 mV pulse intensity Paper speed	Patient Simulator (Fluke MPS450)	7	3	4	14	12 months

Pulse oximeter	ECG BPM test Oxygen saturation	SPO2 Analyzer (Fluke Index 2 XLF)	7	3	4	14	12 months

Sphygmomanometer	Pressure leakage Pressure accuracy	NIBP Simulator (Fluke BP Pump 2L)	6	3	4	13	12 months

Infant incubator	Temperature test Humidity test Noise test Baby probe test	Patient Simulator (Fluke MPS450)	10	5	5	20	6 months

Phototherapy unit	Intensity	Phototherapy Analyzer (Dale 40)	8	4	5	17	6 months

Defibrillator	ECG BPM test ECG amplitude test ECG arrhythmia test Energy test Charge time test Sync. discharge test	Defibrillator Analyzer (Fluke QED 6H)	10	5	5	20	6 months

Aspirator	Max. free flow Rate of vacuum rise Max. vacuum Vac. gauge accuracy	Flow Analyzer (Fluke VT Plus)	9	3	4	16	12 months

Electrosurgical unit	Cutting-power test Coag. power test Bipolar-power test HF leakage test REM alarm test	Electrosurgical Unit Analyzer (Rigel UNITHERM)	9	5	5	19	6 months

**Table 3:** Data list screen (8 records; total: 589 records).

Location	Device name	Brand code	Serial number	Status	Error
ECG room	ECG	Nihon Kohden	4971	Passed	No problem
Service	SPO2	Massimo	N43378	Passed	No problem
Emergency	Sphygmomanometer	Riester	091250123	Failed	High pressure leakage
Infant intensive care unit	Infant incubator	Fanem	CI1649	Failed	High temperature
Infant intensive care unit	Phototherapy unit	Medix	560-09	Passed	No problem
Emergency	Defibrillator	Nihon Kohden	07728	Failed	Low battery
Operation room	Aspirator	Bıçakcılar	1598	Failed	Low vacuum
Operation room	Electrosurgical unit	Martin	BO 88 74	Failed	Power circuit error

In total, the data of 589 medical devices were listed.

**Table 4:** Predictive maintenance time schedule including newer high-tech devices.

Predictive maintenance time schedule								
Device	Brand	Model	Daily	Every	Every	Every	Every	Every
				1 m	3 m	4 m	6 m	12 m
CT	Philips	Brilliance CT 16-Slice	×			×		
	Siemens	Somatom Sensation 4	×			×		
	Toshiba	Aquilion 64	×		×			

Angio	Philips	MultiDiagnost Eleva	×			×		
	Siemens	Axiom Artis dtA	×			×		

Mammo	IMS	Giotto SDL	×			×		

C-arm	Siemens	Arcadis Varic	×				×	
	Siemens	Arcadis Varic Gen 2	×				×	
	Siemens	Siremobil Compact	×				×	
	Siemens	Siremobil Compact L	×				×	
	Philips	BV Endura	×				×	

MR	Siemens	Magnetom Symphony	×			×		
	Philips	Achieva 1,5 T	×			×		
	Philips	Achieva 3,0 T	×			×		

Pet-CT	Siemens	Biograph 6 TruePoint	×		×			

Gamma	Siemens	E-Cam Extended Gantry	×		×			×
Camera	Mediso	Nucline DHV-2 Sprit	×	×	×		×	×
	Mediso	Nucline TH-22	×	×	×		×	×

Ventilator	Maquet	Servo-s	×				×	
	Maquet	Servo-i	×				×	
	Draeger	Babylog 8000 plus	×				×	
	Draeger	Evita 4 Neoflow	×				×	
	GE	Engström	×				×	

Anesthesia unit	Draeger	Fabius	×				×	
	Draeger	Fabius GC	×				×	
	Draeger	Julian	×				×	
	Draeger	Primus	×				×	
	GE Datex	Avannce S5	×				×	
	GE Datex	Aestiva 5	×				×	

Istanbul University Hospitals.

Predictive Maintenance Program 2014.

**Table 5:** Medical device failures.

Medical device	Total #	Number	Errors	Error code
Electrocardiogram	50	2	Not working	ECG01
		1	Power circuit error	ECG02
		1	Paper speed error	ECG03
		3	Electrode error	ECG04
		1	Sensitivity error	ECG05

Pulse oximeter	46	3	Not working	SPO01
		1	Low BPM	SPO02
		1	High BPM	SPO03
		2	Low oxygen saturation	SPO04
		2	High oxygen saturation	SPO05
		5	Probe error	SPO06

Sphygmomanometer	200	12	Not working	SPG01
		15	High pressure leakage	SPG02
		8	Cuff error	SPG03
		4	Broken manometer	SPG04
		5	Missing piece	SPG05

Infant incubator	28	3	Not working	INC01
		2	Over temperature	INC02
		1	Display error	INC03
		1	Baby probe error	INC04
		2	Broken cover	INC05

Phototherapy unit	30	3	Not working	PHT01
		2	Low intensity	PHT02

Defibrillator	86	3	Not working	DEF01
		1	Low/high energy	DEF02
		3	Low battery	DEF03
		2	Electrode error	DEF04
		1	Paddle error	DEF05
		2	BPM error	DEF06
		2	Synchronization error	DEF07

Aspirator	97	6	Not working	ASP01
		4	High vacuum	ASP02
		3	Low vacuum	ASP03
		3	Maximum vacuum	ASP04
		2	Vacuum rise error	ASP05

Electrosurgical unit	52	1	Not working	ESU01
		3	Power circuit error	ESU02
		3	High/low cut power	ESU03
		2	High/low coag. power	ESU04
		1	High/low bipolar power	ESU05
		2	Foot switching error	ESU06
		2	Patient electrode error	ESU07

**Table 6:** Preventive maintenance time schedule including older technology devices.

Preventive maintenance time schedule							
Device		Daily	Weekly	Every	Every	Every 12 m	As needed
				3 m	6 m		
Infant incubator	Baby temperature probe			×			
	Air filter			×			
	Electrical fuse			×			
	Switch			×			
	Breaker relay			×			
	Fan motor				×		
	Vacuum compressor motor				×		
	Pressure sensor				×		
	Noise sensor				×		
	Air circulation sensor				×		
	Humidity sensor				×		
	Temperature sensor				×		
	Accumulator	×					
	Gasket						×

Aspirator	Air input filter			×			
	Pump motor			×			
	Fan motor			×			
	Air hose		×				
	Fluid suction hose	×					

Defibrillator	Battery		×				
	Spoon connection cable		×				
	Fibrillation detection sensor				×		
	Charging transformer						×
	Heart beat sensor				×		
	ECG sensor				×		
	Leakage relay			×			
	Electrical fuse			×			
	Defibrillation time sensor				×		

Pulse oximeter	Probes			×			
	Optic sensor				×		
	Battery		×				
	Connector						×

Istanbul University Hospitals

Preventive Maintenance Program 2014.
